# Difficulties in the Surgical Diagnosis and Treatment of Cases Associated with Vomiting in Children

**Published:** 1906-06

**Authors:** James Swain

**Affiliations:** Professor of Surgery in University College, Bristol; Surgeon to the Bristol Royal Infirmary


### DIFFICULTIES IN THE SURGICAL DIAGNOSIS AND TREATMENT OF CASES ASSOCIATED WITH VOMITING IN CHILDREN.

BY

JAMES SWAIN, M.S., M.D.LOND., F.R.C.S.,

*Professor of Surgery in University College, Bristol; Surgeon to the Bristol Royal Infirmary.*

---

VOMITING, in association with pain and collapse, is found in almost every case of acute abdominal crisis, whatever the cause of the mischief may be—appendicitis, perforated gastric ulcer, a twisted ovarian pedicle, the passage of a renal calculus, and numerous other conditions, are all characterised in the early stage by this triad of symptoms, for which Treves suggested the term "peritonism." Under such circumstances we have not to wait long, as a rule, before the localisation of pain and tenderness lead to a sufficient diagnosis of the case for the purposes of treatment.

If the vomiting is produced by one of the many forms of intestinal obstruction, the retracted and rigid abdomen of the beginning of the attack is soon replaced by a gradually increasing abdominal distension, and the same is true of many cases of perforative peritonitis. It is not desirable to wait for this abdominal distension in cases where the diagnosis is clear, and I have always found it to be a safe surgical practice to open the abdomen without delay in cases of doubtful diagnosis, where the vomiting is associated with a gradually increasing tympanites.

All rules, however, have their exceptions, and, notably in the case of obstruction due to intussusception, meteorism is generally absent; but the diagnosis of many of these cases is made sufficiently evident by the existence of a tumour, which hardens under the hand in association with attacks of pain, and the passage of bloody mucus.

The absence of abdominal distension in most cases of intussusception is, however, a factor in the difficulty of diagnosis in cases associated with vomiting in children, and if it is borne in mind that a tumour cannot be felt in more than half the cases of intussusception and that the passage of blood-stained mucus is absent in twenty per cent., it will be apparent that many conditions may lead to confusion, either by causing us to overlook an existing intussusception, or to wrongly assume the presence of an invagination in cases of vomiting due to other causes.

As an exemplification of these remarks, it may be mentioned here that in two of the cases recorded below it was thought probable that one of these anomalous cases of intussusception existed. An actual intussusception was found in one of them, but it is doubtful whether it was the cause of the vomiting.

Two of the three cases to which I wish to refer may be now stated, as they appear to have much in common; the third case probably comes in another category.

**Case I.** A. S., male, aged 4 years, after two or three days' malaise, was seized with frequent vomiting, which continued for twelve days before I saw him. The vomiting was almost the only symptom throughout, and at first was more severe than it had been for the past few days. Some tenderness had been noticed over the liver for four days. The temperature remained normal except on one occasion, when it was found to be 99.4. The bowels had acted regularly, but at first there was a little diarrhœa. There was no family history of tubercle. Considering the duration of the vomiting emaciation was not marked. The child was very apathetic, and the face had a drawn, anxious look. There was no complaint of pain, but there was distinct tenderness over the liver, which was enlarged. The abdomen was flat. An exploratory incision revealed some congestion of the small gut, and in the mesentery numerous enlarged lymphatic glands were found varying in size from a pea to a grape. These were of firm consistence and yellowish white in colour ( ? from early caseation). Nothing else abnormal was noticed and the abdominal wound was closed. No further sickness occurred and the child made a rapid recovery, and at the end of a month was "fat and chubby."

**Case II.** D. B., female, aged 5 years, after a period of twenty-four hours, in which she complained of feeling ill, was seized with vomiting which had lasted for six days before I saw her. For five days practically no food had been taken, as it was found that only by diminishing the food could the vomiting be checked. Only on one or two occasions had there been a complaint of slight abdominal pain. The bowels had acted spontaneously and after an enema, and the motions contained more mucus than fæces, but no blood. The temperature was normal throughout, except for an occasional rise to 99°. The pulse (110) had been gradually rising. There had been no abdominal distension. No family history of tubercle. The child looked very ill with a slight icteric tint of the conjunctivæ. There was a peculiar odour about the breath. The abdomen was retracted. The liver was enlarged, and apparently below it an ill-defined swelling (afterwards thought to be due to the enlarged liver itself) was felt near the hepatic flexure of the colon. The case was regarded as possibly one of anomalous intussusception and an explanatory cœliotomy advised. On opening the abdomen, the small intestine was seen to be empty and congested, and numerous enlarged (up to the size of a grape) mesenteric glands without signs of caseation were present. Nothing else abnormal was found, and the abdomen was closed after filling it with saline solution. The vomiting continued, and the child died about thirty-seven hours after operation. A *post-mortem* examination was made by a skilled pathologist, who found the liver enlarged and fatty. There were numerous enlarged mesenteric glands, and the solitary follicles of the big gut were enlarged. All other organs were normal. An examination of the urine revealed the presence of acetone.

Case I. was operated upon some years ago, and the presence of acetone in the urine was not thought of, but its existence in Case II. led to the suggestion that the disease might be one of the class included under the designation of "cyclical" or "periodic vomiting." Whatever the correct diagnosis of these cases may be, it is evident that they both bear a marked resemblance to this comparatively new disease. In each instance there was a prodromal stage followed by vomiting, apparently without cause and unaccompanied by any marked degree of temperature or abdominal pain. In Case II. there was also a peculiar odour of the breath and acetonuria, and I subsequently learnt that the child had had attacks of sickness before.

I have examined the urine of cases suffering from hip disease, burns, and other conditions taken indiscriminately, and have always found evidence of the existence of small quantities of acetone, so that—as pointed out by Von Jaksch—it may be regarded as a normal constituent of the urine. The withdrawal of carbo-hydrates from the diet, fasting and other conditions, appear to increase the amount of acetone in the urine, so that in such cases as these we have been considering it seems difficult to decide whether the acetonuria is an effect or a cause of the vomiting; but the fact that an excess of acetone has been found in the urine in the early stage of the disease lends support to the opinion of some observers that acetonuria is the causative factor in cyclical vomiting.

The chief point of interest to the surgeon is how to recognise cases of cyclical vomiting from those anomalous forms of intussusception, to which reference has already been made.

The history of previous attacks, if it exists, would certainly be helpful, but this would not be available in a first attack. The smell of the breath, the absence of pain and the presence of a large quantity of acetone in the urine would probably make one hesitate to perform an operation before trying the effect of rectal feeding and the administration of massive doses of the alkaline carbonates if time allowed; but where (as in these cases) the child seems likely to die unless speedily relieved, it is doubtful whether it would be wise to adopt any other course than that of an exploratory cœliotomy. Cases of cyclical vomiting sometimes end fatally, and the risk of an exploratory incision is so slight that it is probable that, in the event of death following on such an operation undertaken for diagnostic purposes, the fatal issue is due rather to a continuance of the disease than to the surgical procedure.

Cyclical vomiting as now understood should be independent of dietetic error and unaccompanied by pain, except such as may be due to the excessive vomiting, and from the surgical point of view the absence of marked pain is perhaps the most important clinical fact in the two cases just considered, for pain is generally a prominent symptom in intussusception.

Where pain exists in conjunction with vomiting, the difficulty of diagnosis is more marked in the absence of the usual signs of intussusception. This is seen in the following case.

**Case III.** J. N., male, aged 4 years. Five days before I saw him the patient was said to have eaten a good many raw peas, and this was followed by pain in the abdomen. Two days later he vomited and complained of much pain in the umbilical region, and since then diarrhœa and vomiting had been more or less frequent. Practically no solid fæces had been passed, but the motions consisted chiefly of blood-stained mucus mixed with soft fæcal matter, and on one occasion he passed green peas in the stool. On the day of examination he was much worse, the pain had increased, and the temperature had risen to 102°. The pulse was 156 and the respirations 36 per minute. The boy looked very ill, but there was no distension of the abdomen, no tenderness and no lump to be felt. The rectum was empty. An exploratory cœliotomy was advised, and the patient was admitted to the Royal Infirmary under the care of one of my colleagues. Green peas were found in the vomit after admission. On opening the abdomen a small intussusception (half an inch) was seen in the small gut. This at once released itself, but the absence of swelling or distension in the neighbourhood led to the belief that the symptoms were in no way connected with the intussusception. The patient died the following day, and at the *post-mortem* examination nothing special was found except some intestinal congestion, and the general feeling seemed to be that the cause of the mischief was a form of gastro-enteritis set up by some irritant (? peas). Acetone was present in the urine.

The abdominal pain and passage of bloody mucus in this case were highly suggestive of an intussusception, though the flaccid abdomen and the history of pain following on the ingestion of raw peas, which subsequently appeared in the vomit and fæces, led to an alternative diagnosis of gastro-enteritis. There seemed no other way of deciding the point except by an abdominal exploration, for the patient was getting rapidly worse. The acetonuria was probably an effect of the vomiting.

Such cases as those to which reference has been made present much difficulty in diagnosis, and it seems necessary at times to resort to cœliotomy in order to clear up the matter; but in these several conditions associated with vomiting, in which the child seems progressing from bad to worse, it is surely better to err occasionally in opening the abdomen in a case where surgical help is not strictly applicable, than to omit the performance of an operation in an anomalous case of intussusception or other form of obstruction.

By an examination of the urine for excess of acetone and by closer attention to the points I have endeavoured to illustrate, we may hope in some cases to arrive at a probable diagnosis sufficiently early to allow of a trial of certain definite measures before the condition of the patient becomes so critical that there is only time to effect a diagnosis by abdominal exploration.

